# GPX4‐mediated bone ferroptosis under mechanical stress decreased bone formation via the YAP‐TEAD signalling pathway

**DOI:** 10.1111/jcmm.18231

**Published:** 2024-03-17

**Authors:** Wang Mengjia, Ji Jun, Zhang Xin, Zhang Jiahao, Guo Jie

**Affiliations:** ^1^ Department of Orthodontics, School and Hospital of Stomatology, Cheeloo College of Medicine, Shandong University & Shandong Key Laboratory of Oral Tissue Regeneration & Shandong Engineering Research Center of Dental Materials and Oral Tissue Regeneration & Shandong Provincial Clinical Research Center for Oral Diseases Jinan Shandong China; ^2^ Department of Orthodontics Nanjing Stomatological Hospital, Affiliated Hospital of Medical School, Research Institute of Stomatology, Nanjing University Nanjing China

**Keywords:** ferroptosis, gpx4, mechanical force, osteoblasts

## Abstract

Fracture of the alveolar bone resorption is a common complication in orthodontic treatment, which mainly caused by extreme mechanical loading. However, the ferroptosis with orthodontic tooth movement(OTM) relationship has not been thoroughly described. We here analysed whether ferroptosis is involved in OTM‐associated alveolar bone loss. Mouse osteoblasts (MC‐3T3) and knockdown glutathione peroxidase 4 (GPX4) MC‐3T3 were stimulated with compressive force loading and ferrostatin‐1 (Fer‐1, a ferroptosis inhibitor), and the changes in lipid peroxidation morphology, expression of ferroptosis‐related factors and osteogenesis levels were detected. After establishing the rat experimental OTM model, the changes in ferroptosis‐related factors and osteogenesis levels were reevaluated in the same manner. Ferroptosis was involved in mechanical stress regulating osteoblast remodelling, and Fer‐1 and erastin affected osteoblasts under compression force loading. Fer‐1 regulated ferroptosis and autophagy in MC‐3T3 and promoted bone proliferation. GPX4‐dependent ferroptosis stimulated the YAP (homologous oncoproteins Yes‐associated protein) pathway, and GPX4 promoted ferroptosis via the YAP‐TEAD (transcriptional enhanced associate domain) signal pathway under mechanical compression force. The in vivo experiment results were consistent with the in vitro experiment results. Ferroptosis transpires during the motion of orthodontic teeth, with compression force side occurring earlier than stretch side within 4 h. GPX4 plays an important role in alveolar bone loss, while Fer‐1 can inhibit the compression force‐side alveolar bone loss. GPX4's Hippo‐YAP pathway is activated by the lack of compression force in the lateral alveolar bone.

## 1 INTRODUCTION

The objective of orthodontic tooth motion is to attain equilibrium between osteoblasts and osteoclasts. It is of two types: compression force side and tension side. It causes bone formation and absorption. The production of active oxygen by reactive oxygen species (ROS) leads to an imbalance in osteoblasts and bone metabolism, resulting in a powerful oxidative stress resistance system. Both sides of the equation are affected by osteoblasts, with a decrease in compression force and an increase in tension. Oxidative stress can lead to cell apoptosis, which can lead to an increase in lipid peroxidation and a decrease in the osteogenesis of bone marrow mesenchymal stem cells, thus diminishing bone mass and formation.^1^, ^2^, ^3^, ^4^

Treatment of orthodontics is a necessity, particularly for female adults. The application of orthodontic force within the physiological range, especially in patients with osteoporosis, is more likely to cause alveolar bone resorption retraction, that is ‘black triangle’ generation, causing adverse impacts in patients. Bone destruction, resulting in harm to bone remodelling, renders orthodontic treatment ineffective, thus adversely impacting the prognosis of such treatment.

A metabolic death type recently identified, ferroptosis is distinguished by the close relationship between lipid peroxidation and iron metabolism, which results in bone growth.^5^, ^6^, ^7^

The key to the equilibrium between inducing cell death and sustaining cell stability lies in the control of ferroptosis and intracellular iron levels, which are the foundation of biological operation and are essential in the early identification of musculoskeletal issues like osteoporosis. In models of iron‐overload that lead to accelerated bone deterioration, bone remodelling is defined by the formation and absorption of bone. At the peak of bone formation in young adults, mineral density reaches its zenith between 25 and 30 years of age; however, postmenopausal women^8^, ^9^ experience a decline in bone mineral density from 30 years of age. Iron accumulation in bone increases with age and causes bone loss. Recent studies have shown that iron metabolism produces significant toxicity during bone mineralization, significantly increasing osteoblast death and decreasing mineralization ability.^5^, ^10^, ^11^, ^12^

GPX4, a central glutathione‐utilizing enzyme, has been found to regulate ferroptosis and inhibit cell death by depleting the glutathione that initiates it.^13^ Molecular compounds such as erastin inhibit the activity of phospholipid hydroperoxide GPX4 and lead to ferroptosis.^14^ Fer‐1 is a selective erastin‐induced ferroptosis inhibitor that inhibits oxidized, iron‐dependent cancer cell death by blocking cystine transport and glutathione production.

Iron first accumulates in osteoblasts and affects their differentiation by significantly downregulating its progression toward the bone matrix. The expression of inflammatory factors was found to be augmented by iron exposure in osteoblasts and osteocytes, resulting in an increase in intracellular iron levels.^15^ Iron death is a result of augmented oxidative stress due to the high iron concentration in bone, leading to the demise of osteoblasts and osteocytes.^16^ This process is conducive to the generation and recruitment of osteoclasts, thus leading to an imbalance in bone metabolism.^17^ Previous studies reported an association between iron overload and osteoporosis, and that transient iron overload could lead to complete inhibition of bone metabolism and imbalance in bone formation and resorption.^18^, ^19^, ^20^ Thus, ferroptosis may be involved in tissue degradation when the iron levels are too high.

When mechanical stress is applied to bone tissue, osteoblasts and osteoclasts are subjected to the corresponding tensile and compressive stresses, leading to bone destruction and ferroptosis reaction, altering the trabecular arrangement and density, and further inducing pathological bone remodeling. A study revealed that the sensitivity to ferroptosis is greatly impacted by cell contacts and cellular density.^21^

The Hippo pathways, such as YAP and TAZ (transcriptional coactivator with PDZ‐binding motif), are activated, thereby stimulating ferroptosis in cancer cells through the regulation of ACSL4 (acyl‐CoA synthetase long‐chain family member 4).^22^

Previous investigations, however, mainly concentrated on the molecular level and iron toxicity associated with bone depletion. Some researchers believed that iron increased bone resorption^5^, ^10^, ^23^; some studies indicated the inhibition of osteoblast differentiation.^19^, ^24^, ^25^ Iron's toxic influence on bone mineralization is noteworthy, leading to a noteworthy rise in mortality of osteoblasts. Therefore, the interaction between iron metabolism and bone metabolism was more obvious. The objective of this research was to explore how stress‐induced ferroptosis disrupted the equilibrium between bone reabsorption and formation.

## 2 MATERIALS AND METHODS

### 2.1 Cell culture

The MC‐3T3‐E1 mouse preosteoblast precursor cells, purchased from Cyagen Oricell, a product line of Cyagen Biosciences Inc., were used in the experiments. These cells were designated for osteoblast and bone‐specific protein (BSP, OCN) mRNA studies. The cell culture was maintained in α‐MEM medium (sourced from Gibco BRL, MD, USA), enriched with 10% foetal bovine serum obtained from Pan‐Biotech, Aidenbach, Germany, and the medium was refreshed bi‐daily. The differentiation of these cells into mature osteoblasts was stimulated by supplementing the culture medium with ascorbic acid (concentration of 50 mg/L) and β‐glycerophosphate (at a concentration of 5 mM). This process necessitated routine subculturing at 4‐day intervals. All experimental procedures were carried out under conditions favourable for osteoblast induction.

### 2.2 Cell compressive force model

Initially, round glass panes (30 mm in diameter, 4 mm thick, and with a density of 2.5 × 10^3^ kg/m^3^) were customized and sterilized. In each well of a 6‐well plate, MC‐3T3 osteoblasts were seeded at a density of 2.0 × 10^5^ cells. To simulate compressive pressure of 1 g/cm^2^ on the MC‐3T3 cells, a glass pane was placed over the cells in a single well for durations of 0, 2, 4 and 6 h. For the control group, the cells were subjected to 0 h of pressure.

### 2.3 Micro‐computed tomography (micro‐CT) analyses

The femora of mice were scanned using a SkyScan 11,772 microcomputed tomography system from Bruker, Belgium. Key parameters such as trabecular thickness (Tb.Th), bone volume per tissue volume (BV/TV) and trabecular separation or spacing (Tb.Sp) were analysed in the samples. For reconstructing three‐dimensional images of the mouse distal femora, Mimics 18.0 software, developed by Materialize in Belgium, was utilized.

### 2.4 Chemicals and antibodies

Sigma‐Aldrich (MO, USA) supplied Fer‐1 and erastin, while Proteintech Group, Inc. provided the primary antibodies: anti‐YAP (20536‐1‐AP,1:1000), anti‐RUNX2 (AF5186,1:1000; Affinity Biosciences), anti‐TEAD2 (21159‐1‐AP,1:1000; Proteintech Group, Inc.), Lamin B1 antibody (12987‐1‐AP,1:1000; Proteintech Group, Inc.), anti‐β‐catenin (20536‐1‐AP,1:1000; Proteintech Group, Inc.), anti‐GPX4 (ab125066,1:1000; Abcam) and anti‐ACSL4 (ab177958,1:1000; Abcam). Cell Signaling Technology (MA, USA) supplied all the secondary antibodies.

### 2.5 Immunofluorescence assay

After washing the cells separated in a six‐well plate with phosphate‐buffered saline (PBS), they were incubated in a blocking solution containing paraformaldehyde for 1 h. Subsequently, the cells were incubated with the primary rabbit anti‐GPX4 (1:100) and anti‐ACSL (1:100) antibodies at 4°C for a further 1 h. Subsequently, the cells were washed with PBS three times and DAPI (4′,6‐diamidino‐2‐phenylindole) was added at room temperature for 10 min. Under a fluorescent microscope (Axio Imager M2, Zeiss, Germany), they were then observed.

### 2.6 GPX4 knockdown in MC‐3T3‐E1 cells by lentivirus

Three distinct sequences for the lentivirus, inclusive of sgRNA sequences, (GPX4‐RNAi, target seq:atCGATCTGCATGCCCGATAT) were synthesized by Sangon Biotechnology, located in Shanghai, China. The LentiCRISPRv2 vector was kindly provided by Brett Stringer (Reference: Addgene_98,290; available at net/addgene:98290). We generated lentiviral vectors that express sgRNAs targeting GPX4, in line with methodologies described in previous studies.^26^, ^27^ The accuracy of these constructs was confirmed through DNA sequencing. Subsequently, 293 T cells were used for transfection. These cells produced lentiviral particles that were then employed to infect the MC‐3T3‐E1 cells. To assess the effectiveness of GPX4 knockout in MC3T3‐E1/GPX4KO cells, immunoblotting was conducted. The selected cells were maintained in a medium containing 2.0 μg/mL puromycin (sourced from Sigma‐Aldrich) for a duration of 7 days to ensure stability.

#### 2.6.1 Reverse transcription‐quantitative polymerase chain reaction

Total RNA from MC‐3T3 cells was extracted using TRIeasy Total RNA Extraction Reagent, provided by Yeasen Biotechnology in Shanghai, China. This RNA was then converted to cDNA using the Hifair III First‐Strand cDNA Synthesis SuperMix, suitable for quantitative polymerase chain reaction (qPCR) with gDNA digester plus (also from Yeasen). The RT‐qPCR was performed using a CFX96 RT‐PCR Detection System from Bio‐Rad, CA, USA, and the Hieff qPCR SYBR Green Master Mix (Low ROX Plus) by Yeasen. Primer sequences are listed in Table 1. The qPCR cycling protocol was in accordance with the method described by Li et al. (2021). Relative mRNA expression levels were quantified using the comparative 2^−ΔΔCt^ method and normalized against the internal control gene, Glyceraldehyde‐3‐phosphate dehydrogenase (GAPDH).

**TABLE 1:** Primer table.

GPX4	CCCGATATGCTGAGTGTGGTTTA	TTCTTGATTACTTCCTGGCTCCTG
Acsl4	GCCATGGAAGCTGAAATACTGAAAG	GAAGGCATCTGTTACCAAACCAGTC
FTH1	CATCAACCGCCAGATCAACCT	GCAAAGTTCTTCAGAGCCACATCA
Tead1	GAGCGACTCGGCAGATAAGC	CCACACGGCGGATAGATAGC
Tead2	GAAGACGAGAACGCGAAAGC	GATGAGCTGTGCCGAAGACA
Tead3	GTCCAGCCACATACAGGTTCT	GGAGACTTGGTCCAGGTTCATAG
Tead4	TCCGCCAAATCTATGACAAGTTC	CGATGTTGGTATTGAGGTCTGC
YAP1	CCGGGATGTCTCAGGAATTG	CTGTAGCTGCTCATGCTTAGTCCA
CTNNB1(beta‐catien)	GGACCACAAGCAGAGTGCTGA	TTCTGAACAAGACGTTGACTTGGA
Runx2	AACCAAGAAGGCACAGACAGAAG	GGCGGGACACCTACTCTCATA
OPN	GCAGTATGAATTGAATCGGAACAAC	ATGGCCTGGTCCATCTCCAC

### 2.7 Western blot analysis

Protein extraction from bone tissue was carried out using a kit from Beijing Applygen Science Biotechnology, Beijing, China. For total protein extraction from cells, RIPA lysis buffer was used, which was combined with phenyl methane sulfonyl fluoride (PMSF), protein inhibitors and phosphatase inhibitors, all sourced from KeyGen Biotechnology in Nanjing, China. Equal quantities of protein were separated using 10% SDS‐PAGE (sodium dodecyl sulfate‐polyacrylamide gel electrophoresis). The proteins were then transferred to a PVDF membrane, provided by Beytime Biotechnology, Shanghai, China. This membrane was blocked at room temperature with 5% nonfat milk in Tris‐buffered saline with Tween 20 (TBST) for 1 h. For primary antibody incubation, the membrane was kept in TBST at 4°C overnight. After washing thrice with TBST, the membranes were treated with horseradish peroxidase‐labelled secondary antibody at 37°C for an hour. The protein bands were visualized using an enhanced chemiluminescence detection reagent, and the band intensity was quantified using a densitometer from Bio‐Rad Laboratories, CA, USA, specifically the ChemiDoc XRS X‐Ray Sensitivity system.

### 2.8 The determination of lipid peroxidation and nitric oxide production

After treatment with MC‐3T3 as prescribed, 50‐μM C11‐BODIPY (#D3861; Invitrogen, CA, USA) was added and incubated for 1 h. Excess C11‐BODIPY(boron‐dipyrromethene) was removed by washing the cells twice with PBS. After trypsinization, the labelled cells were resuspended in PBS and 5% foetal bovine serum. The oxidation of C11‐ BODIPY caused a shift in the fluorescence emission peak from 590 to 510 nm, which was proportional to lipid ROS generation, and was then analysed with a flow cytometer. To measure nitric oxide (NO) production in mouse hearts and MC‐3T3, the total nitric oxide assay kit (#S0021S, Beyotime, Shanghai, China) was used to quantify its stable metabolite (nitrite). A full‐wavelength multifunctional microplate reader JS‐THERMO Varioskan Flash (Thermo Fisher Scientific, USA) was employed to measure the absorbance.

### 2.9 Histological analysis, fluorescence staining and ultrastructure observation

Immunofluorescence staining for GPX4 (#67763‐1‐lg) and ACSL (#16396‐1‐AP), both sourced from Proteintech, PA, USA, was conducted on MC‐3T3 cells according to the protocols provided by the manufacturer. The process included rehydration of the cells, antigen retrieval and blocking with 1.5% BSA. Paraffin‐embedded sections of 5‐μm thickness were then incubated with primary antibodies targeting GPX4 and ACSL at 4°C overnight. After washing with PBS, these sections were incubated with Alexa Fluor 488 or 594 conjugated secondary antibodies (#SA00013‐1/4; Proteintech, IL, USA), followed by nuclear staining with DAPI (#D21490; Invitrogen). These stained sections were observed under an IX51 fluorescence microscope from Olympus, Tokyo, Japan. In addition, mouse maxillary muscle samples, initially fixed in 2.5% glutaraldehyde, were washed with PBS, postfixed with 1% osmium tetroxide for 1 h, and stained with 2% aqueous uranyl acetate. Following a dehydration process in graded ethanol series, the samples underwent infiltration and polymerization. Ultrathin sections were then cut, treated with xylene vapour to flatten, and placed on nickel grids. Imaging of these samples was performed using a JEM‐1400 Plus scanning transmission electron microscope from JEOL, Japan.

#### 2.9.1 Experimental rat orthodontic tooth movement model

Randomly dividing 20 male Wistar rats (weight: 180–220 g) into four groups—control, compression force‐loading, Fer‐1‐treated and Fer‐1‐treated within‐compression force‐loading—the experiment was approved by Shandong University's Institutional Animal Care and Use Committee (No. GD201926). The rats were anaesthetised with 1% pentobarbital sodium (4 mL/kg of body weight). The tension springs (50 g force) were situated between the left maxillary first molars, and the compression force‐loading group was formed around the front teeth. Fer‐1 was injected into the gingival sulcus of the first molar every 2 days for two consecutive weeks. Additionally, no tension spring or Fer‐1 was used in the control group. After 4 weeks of soft‐diet breeding, the rats were put to death.

#### 2.9.2 Measurement of intracellular ROS

A ROS assay kit (Beyotime) was employed to evaluate the ROS concentrations in HGFs. Briefly, MC‐3T3 were seeded on six‐well culture plates (1 × 105 cells/well). Following attachment and starvation, MC‐3T3 were treated with compression force and Fer‐1 for 4 h. After incubation with 10 μmol/L dichloro‐dihydro‐fluorescein diacetate (DCFH‐DA) at 37°C in the dark for 30–40 min, MC‐3T3 were stained with 10 μg/mL 4′,6‐diamidino‐2‐phenylindole dihydrochloride (Solarbio, Beijing, China) for 10 min. Subsequently, the cells were washed thrice with a serum‐free medium to eliminate the DCFH‐DA, and observed under a fluorescence microscope (Olympus).

### 2.10 Protein spectrum analysis and bioinformatics analysis

Proteins from MC‐3T3 cells, specifically those with stable infection by MC‐3T3 LV‐GPX4, were harvested from 10‐cm dishes. The extraction process involved the use of RIPA lysate from Invitrogen, USA, followed by immediate snap‐freezing in liquid nitrogen. Gene Ontology (GO) analyses were performed, categorizing findings into three main groups: biological process (BP), cellular compartment (CC) and molecular function (MF). This involved the utilization of various databases and software, including KEGG for identifying key networks associated with previously identified differentially expressed (DE) proteins. Additionally, InterPro software was employed for protein domain enrichment analysis. This software aids in classifying protein sequences into families and identifying significant domains and locations. The significance of the enrichment in DE proteins was evaluated using a two‐tailed Fisher's exact test, with a *p*‐value threshold of less than 0.05 for all analyses. Furthermore, protein–protein interactions were examined using the STRING database.

### 2.11 Osteogenic differentiation

When the MC‐3T3 cells reached 80% confluence, they were transferred to a 6‐well plate containing growth medium. This was followed by a change to osteogenic induction medium (comprising α‐MEM from Sigma–Aldrich, St. Louis, USA, 10% FBS, 0.1 μM dexamethasone, 50 μM ascorbate‐2‐phosphate and 10 mM β‐glycerophosphate) for a duration of 3 days. Post a 7‐day induction period, total RNA extraction was carried out, and qRT–PCR (quantitative real time‐polymerase chain reaction) was performed for assessing the expression of genes related to osteogenesis. The activity of alkaline phosphatase (ALP) was measured using an ALP detection kit from Jiancheng, Nanjing, China, and staining was done using a BCIP/NBT Alkaline Phosphatase Color Development Kit from Beyotime, Shanghai, China. After staining, the sections were scanned for image capture.

### 2.12 Phalloidin

After inoculating MC‐3T3 cells in medium with slides, they were incubated in a constant temperature incubator at 37°C and 5% CO_2_ until 80%–90% full. Subsequently, they were washed twice with 1 × PBS (pH 7.4) preheated at 37°C. Subsequently, they were fixed in a solution of 4% formaldehyde dissolved in PBS at room temperature for 10 min. Finally, the cells were washed with PBS for 2–3 times for 10 min each time. Finally, they were treated with 0.5% Triton X‐100 solution for 5 min.At room temperature, PBS was used to wash the cells for a period of 10 min, 2–3 times each. The cells on the cover glass were covered with 200 μL of Rhodamine labelled ghost pen cyclic peptide working solution, and incubated at room temperature for 30 min away from light. After cleaning the cover glass with PBS for 3 min each, the cell nucleus was re‐stained with a 200 μL DAPI solution of 100 nM concentration for approximately 30 s. The acquired images were then viewed under either a confocal microscope or fluorescence microscope.

### 2.13 Overexpression lentiviral transfection

Infection of MC‐3T3 cells into 6‐well plates at 30% confluence was conducted, and then a 2.0 × 10^5^cells/mL cell suspension was created. Lay 2 mL (4 × 10^5^cells/well) in each well, and lay 16‐well plate. (2) Cells grew in 12‐20 h before transfecting with lentiviral.

Calculation method of virus dosage: (number of cells × MOI value/virogen titre) × 103 = virus dosage (μL) Add polybrene: The final concentration of polybrene in the cell sample was 5 μg/mL. After 12–20 h of virus infection, discard the medium and replace it with 2 mL of fresh medium per well. After 72 h, a final concentration of 2 μg/mL puromycin was added. This puromycin‐fresh medium was replaced every 2–3D. About 2 weeks after drug screening, Western blotting tests were taken.

### 2.14 Statistical analyses

Statistical analyses of all data were conducted using GraphPad Prism 7 software (GraphPad Software Inc., CA, USA). To compare multiple groups, *t* tests, one‐way or two‐way analyses of variance were applied. The mean ± standard deviation of the data was presented, and a *p* value of less than 0.05 indicated a statistically significant difference. All cell‐based experiments were repeated at least thrice.

## 3 RESULTS

### 3.1 Ferroptosis was involved in mechanical stress regulating osteoblast remodelling

#### 3.1.1 GPX4 decreased sharply under compression force in MC‐3T3 osteoblasts

**FIGURE 1:**
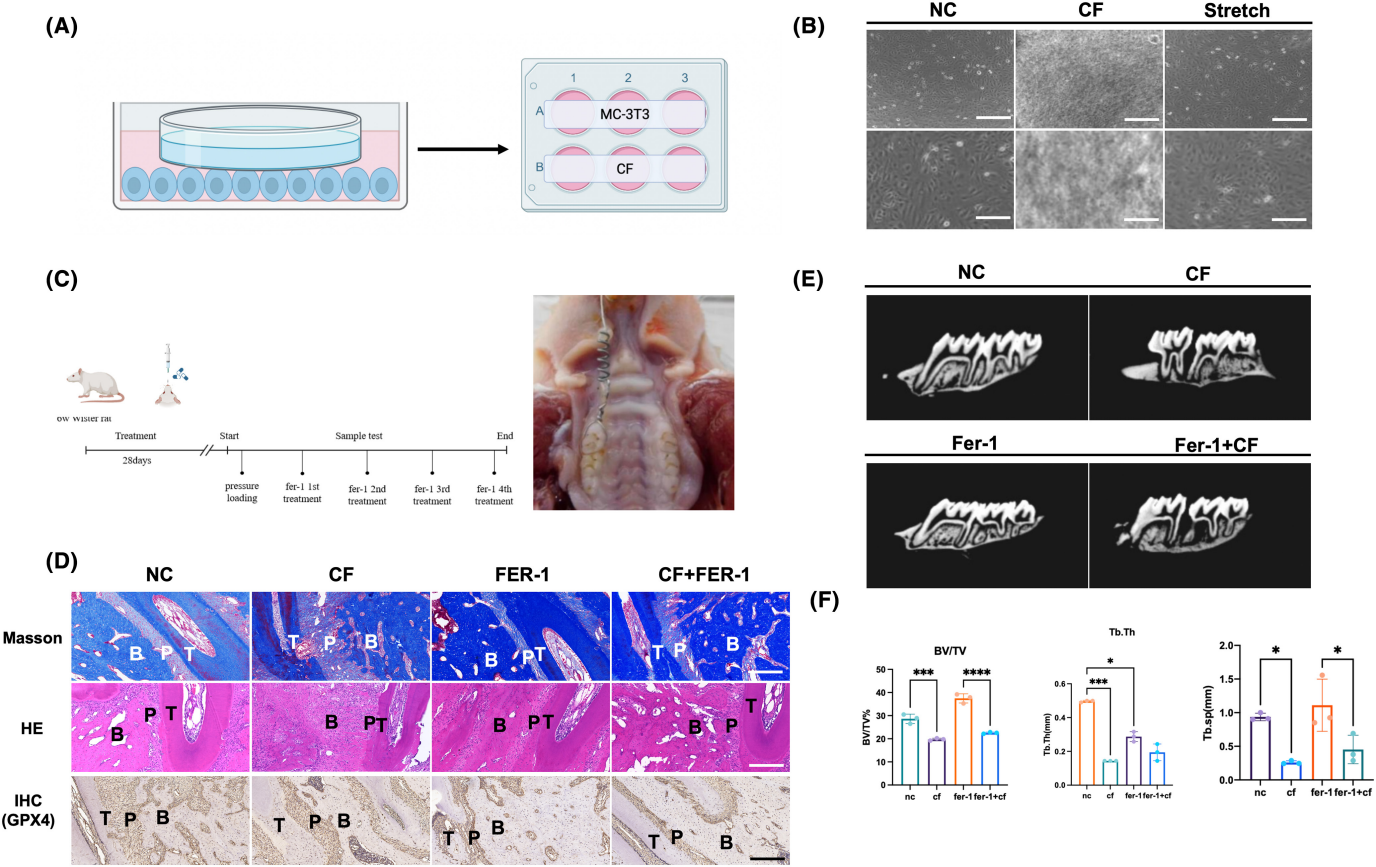
Ferroptosis happens in orthodontics tooth movement (OTM). (A) A 6‐well plate was utilized to cultivate MC‐3T3 cells, and glass panes were applied to osteoblasts in a cell compression force model. (B) The osteoblast was loaded on the tensile side and pressure side. (C) Animal orthodontics tooth movement model constructed. A tension spring was fixed between the rat's first molar and incisor teeth. After 2 weeks, the space was enlarged between the first molar and second molar. (D) Masson stain and immunohistochemistry of GPX4 showed the disorder fibers and constrained vascular endothelial and bone cells in the pressure side. After using ferroptosis inhibitors (Fer‐1), bone formation increased after compression force loading. GPX4 showed different trends in the tensile and pressure side. On the tensile side, GPX4 decreased, while increased on the pressure side, indicating ferroptosis happened on the pressure side. (E) micro‐CT showed the compression force decreased alveolar bone formation, Fer‐1 promoted bone mass increasing (F) schematic diagram of Osteoblast compressive force. A Student's *t*‐test or one‐way ANOVA was conducted to ascertain statistical significance; the error bars symbolize the SD (*n* = 3). *p < 0.05, ***p < 0.001, ****p < 0.0001.

On the pressure side of osteoblasts, cell morphology contractures and increased death were observed, as shown by HE and masson staining (Figure 1A–C). The periodontal membrane was extruded and deformed as alveolar bone was absorbed on the pressure side. Immunohistochemical staining of GPX4 showed a decrease in GPX4 on the stress side, which was reversed with fer‐1, a ferroptosis inhibitor (Figure 1D). Micro‐ct also showed bone changes with a decrease in compressive stress and an increase in bone after fer‐1 treatment of alveolar bone (Figure 1E,F).

#### 3.1.2 Ferrostatin‐1 and erastin affected osteoblasts under compression force loading

**FIGURE 2:**
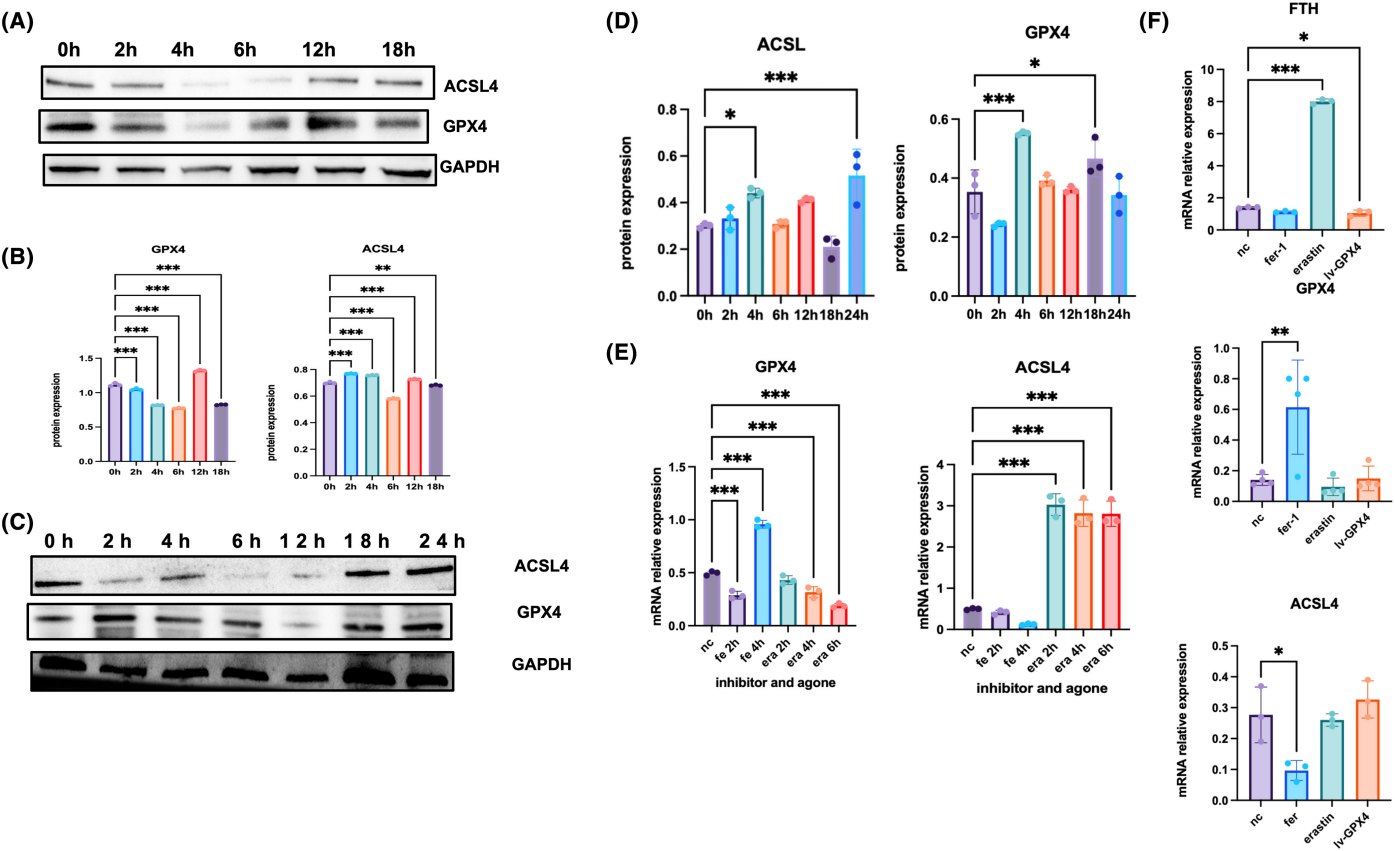
Ferroptosis happened earlier on the pressure side than on the stretch side. (A) On the pressure side, ACSL and GPX4 were significantly expressed in 4 h. ACSL increased compared with GPX4 decreased obviously in 4 h. (B) The ratio of ACSL and GPX4 protein. (C) On the stretch side, ACSL and GPX4 expression changed sharply until 18 h later. (D) the ratio of ACSL and GPX4 protein. (E) Ferroptosis inhibitor and agone treated on MC‐3T3. After treating ferritin‐1 for 4 h, GPX4 decreased sharply, compared with ACSL increased after treatment with erastin in 6 h. (F) After knockdown GPX4 in MC‐3T3, FTH was increased by erastin. GPX4 is expressed highly in fer‐1‐treated cells. ACSL rose up in the lv‐GPX4 group and the erastin group. A Student's *t*‐test or one‐way ANOVA was conducted to ascertain statistical importance. Error bars symbolizing the standard deviation (*n* = 3) were used, and the *p*‐value was less than 0.01. *p < 0.05, **p < 0.01, ***p < 0.001.

Ferrostatin‐1 (Fer‐1) decreased osteoblast ferroptosis and erastin increased ferroptosis under compression force. After the treatment of MC‐3T3 with compression force for 4 h, the GPX4 level decreased sharply, while the ACSL4 level increased within‐compression force loading (Figure 2A,B). After Fer‐1 treatment with compression force loading, the GPX4 level rose up and the ACSL4 level went down. Interestingly, we found that ferroptosis occurred on the compression force side in the MC‐3T3 after 4‐h compression force loading compared with that in the NC (normal control) group. It occurred on the stretch side after 24 h (Figure 2C,D). GPX4 and ACSL4 levels exhibited a significant change in MC‐3T3 under 4‐h compression force loading, while no statistically significant difference was observed in stretch loading. GPX4 level decreased after 6 h compression force loading on the stretch side than on the compression force side, which induced ferroptosis in osteoblasts (Figure 2E,F). These results confirmed that erastin and Fer‐1 affected osteoblast remodelling under mechanical compression force.

#### 3.1.3 Fer‐1 regulated ferroptosis and promoted bone fromation in MC‐3T3

**FIGURE 3:**
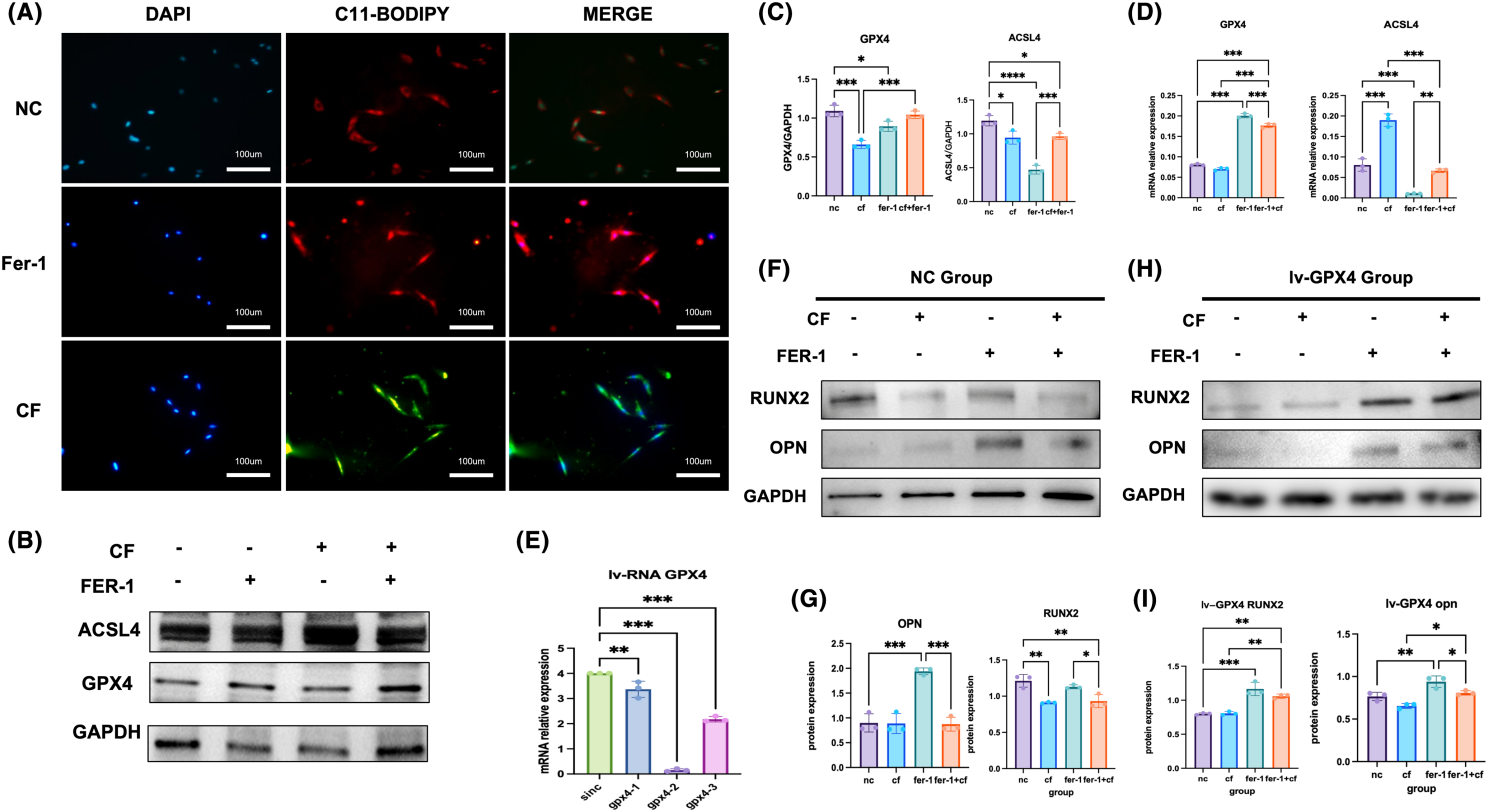
Mechanical compression force‐induced ferroptosis in MC‐3T3 cells. (A) C11‐BODIPY showed a high level of lipid peroxidation in MC‐3T3, and fer‐1 reversed excess lipid peroxidation. (B) Western blotting was employed to evaluate the ACSL and GPX4 protein levels. Pressure caused a rise in ACSL protein and a decrease in GPX4 protein. Fer‐1 inhibited ACSL expression and promoted GPX4 expression.(C–E) The expression of ACSL and GPX4 in both the gene and protein levels can be quantified, as well as the gene expression of GPX4 and ACSL, and the transfection efficiency of GPX4 lentivirus (F, H) the osteogenic protein RUNX2 and OPN expression in MC‐3T3 cells and in knockdown GPX4 cells (G, I) quantitative analysis of Western blotting. A Student's *t*‐test or one‐way ANOVA was conducted to ascertain statistical significance, with all error bars indicating the SD (*n* = 3). *p < 0.05, **p < 0.01, ***p < 0.001, ****p < 0.0001.

Compression force decreased ACSL and GPX4 levels in MC‐3T3 than in the NC group. The GPX4 mRNA level increased after Fer‐1 treatment, and the ACSL level decreased significantly (Figure 3B,C). We used C11‐BODIPY to examine ferroptosis. Lipid peroxidation was stimulated by compression force loading on MC‐3T3 (Figure 3A). These results indicated that Fer‐1 inhibited ferroptosis with compression force in MC‐3T3. We also found that Fer‐1 promoted osteoblast differentiation. RUNX2 and OPN levels increased in MC‐3T3. When we knockdown GPX4 in MC‐3T3 cells, Fer‐1 also reversed low level of GPX4‐induced bone deformation (Figure 3F–I).

#### 3.1.4 Bioinformatics analysis the differentially expressed proteins

**FIGURE 4:**
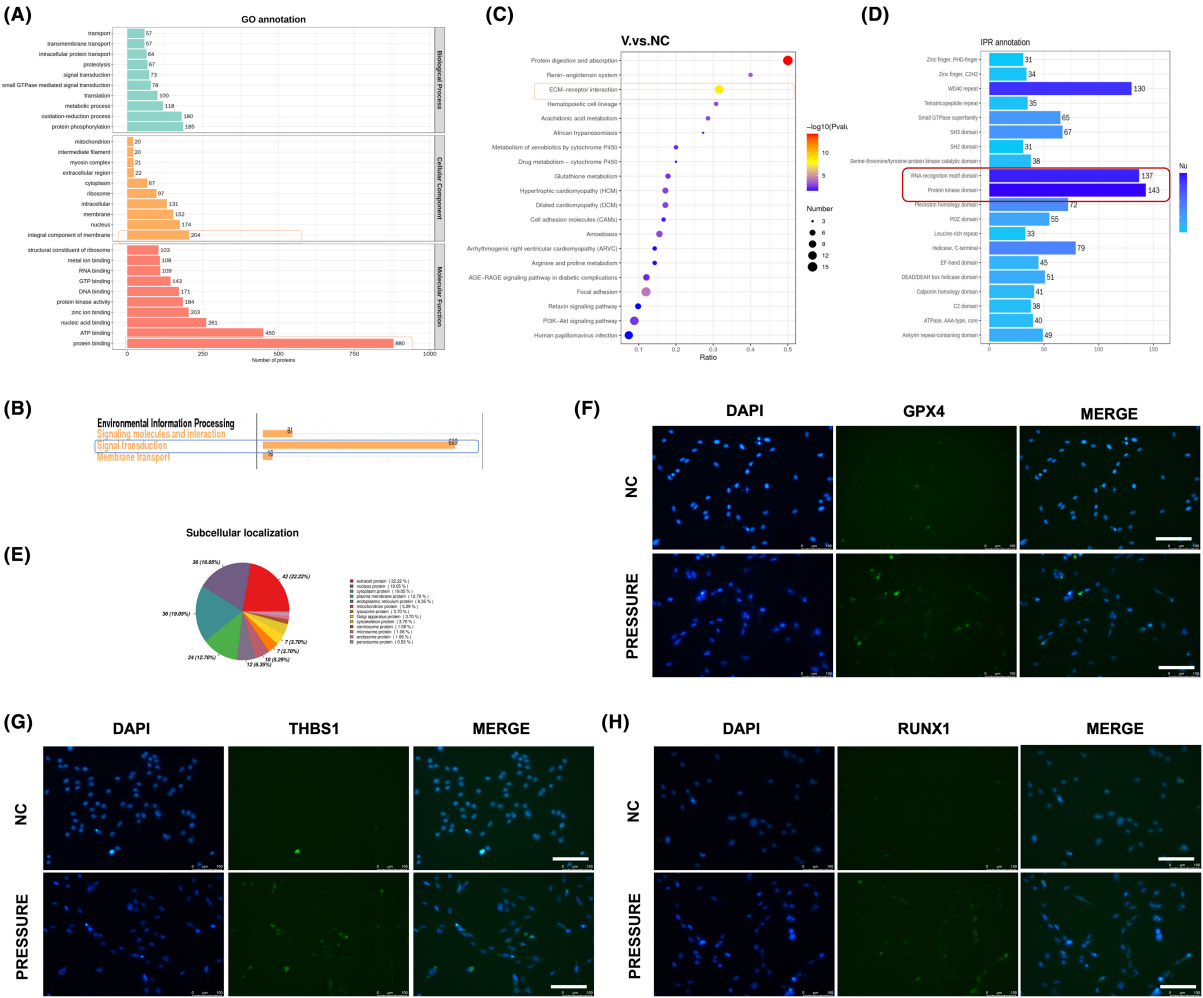
Protein‐sequencing analysis showed that knockdown GPX4 in MC‐3T3 promoted cell transduction between the extracellular matrix and the inner cell. (A) GO annotation data. The component of membrane and protein binding between the normal MC‐3T3 versus knockdown GPX4 group. Note that GPX4 was significantly downregulated were marked in cell transcription, and GPX4 enabled protein crosstalk. (B) COG analysis showed signal transduction significantly increased in environmental information processing by using the protein sequencing data. (C) IPR of the NC and knockdown GPX4 protein sets showed high value in ECM‐receptor interaction. (D) protein kinase domain presented a high value in IPR annotation. (E) Subcellular localization showed GPX4 expressed in the nucleus, cytoplasm and plasma membrane protein. (F–H) Immunofluorescence verification of related increased and decreased proteins. A Student's *t*‐test or one‐way ANOVA was conducted to ascertain statistical importance. All error bars represent the SD (*n* = 3).

Our research primarily concentrated on GPX4 proteins, which, according to bioinformatics analysis, were notably overexpressed. In the context of GPX4 knockdown, the Gene Ontology (GO) analysis revealed that the most expressed proteins, particularly in comparisons between different groups, were associated with ‘integral component of membrane’ and ‘protein binding’ (refer to Figure 4A). Furthermore, we observed a substantial variation in the subcellular localization of these highly expressed proteins across the comparison groups. Notably, in the comparison between the negative control (NC) and the ko‐GPX4 groups, proteins localized in the extracellular space and nucleus constituted nearly a third of the proteins (as shown in Figure 4E). Additionally, the process of environmental information processing also highlighted ‘signal transduction’ as a predominant trend (illustrated in Figure 4B). Intriguingly, the GO analysis indicated a more significant presence of ‘ECM‐receptor interaction’ when comparing the NC and ko‐GPX4 groups (see Figure 4C). Moreover, KEGG pathway enrichment‐based cluster analysis in the ko‐GPX4 group showed a higher enrichment of proteins in the ‘RNA recognition motif domain’ and ‘protein kinase domain pathway’ (depicted in Figure 4D). To further our study, cellular immunofluorescence was utilized to evaluate the expression of THBS1 and RUNX1 under compressive force. It was observed that their expression was elevated compared to the NC group, which aligned with their high expression levels.

#### 3.1.5 GPX4‐dependent ferroptosis stimulated the YAP pathway under compression force

**FIGURE 5:**
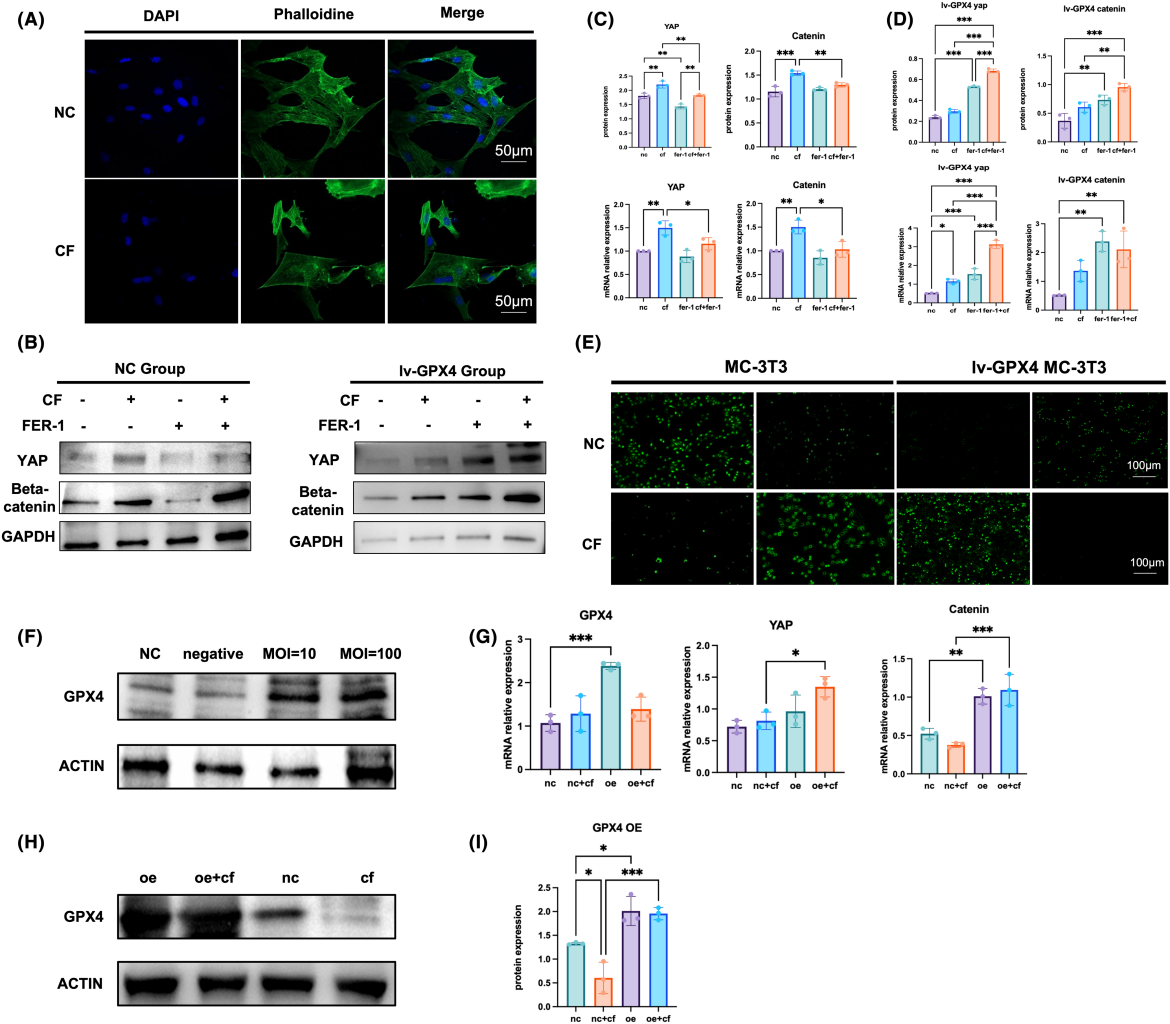
Fer‐1 inhibited mechanical pressure‐induced YAP pathway activation. (A) Phalloidin used confocal microscopy to show cell skeletal disordered and twisted under mechanical pressure. (B) Western blotting presented YAP and β‐catenin expression. YAP level rose in the pressure group and β‐catenin went down after pressure loading. Fer‐1 inhibited YAP expression. (C) the ratio of YAP and β‐catenin at gene level and protein level. (D) Western blotting presented YAP and β‐catenin expression in lv‐GPX4 MC‐3T3 cells. After knocking down GPX4, YAP and β‐catenin levels were significantly enhanced by Fer‐1. (E) ROS level of MC‐3T3 and lv‐GPX4 (F) Overexpression transfection efficiency of GPX4 protein (G) overexpression GPX4 increased YAP and ß‐catenin gene expression in compressive force group (H, I) compressive force promoted GPX4 protein expression in oeGPX4 group. A Student's *t*‐test or one‐way ANOVA was conducted to ascertain statistical importance. All error bars represent the SD (*n* = 3). *p < 0.05, **p ≤ 0.01, ***p < 0.001.

The YAP level increased sharply with compression force loading, while the β‐catenin level decreased. β‐catenin played a significant role in ECM‐integrin reaction and increased with compression force loading and Fer‐1 treatment. We used phalloidin to detect changes in the cytoskeleton under stress, and found that compressive stress‐induced distortion and breakage of the cytoskeleton (Figure 5A), As the signal transduction factor, the kit ligand increased with both compression force loading and Fer‐1 treatment. After GPX4 knockdown in MC‐3T3, YAP could not be activated under compression force, nor β‐catenin (Figure 5B–D). To test whether compressive stress can regulate the YAP pathway through fer‐1, we examined osteoblasts treated with fer‐1 alone and osteoblasts treated with fer‐1 and compressive stress in groups. When treated MC‐3T3 with Fer‐1, YAP and β‐catenin levels increased. The immunofluorescence assay showed that Fer‐1 increased the YAP level and decreased the ACSL and β‐catenin levels. Hence, we confirmed that GPX4‐dependent ferroptosis stimulated the YAP pathway through compression force loading, resulting in cell structure remodelling. ROS assay also confirmed compressive force‐induced cell death. Inhibition of cell death caused by metabolites created by lipid peroxidation can be achieved by Fer‐1. When GPX4 is knocked down, fer‐1 increases reactive oxygen species and promotes osteoblast survival (Figure 5E). To examine the effect of compressive stress on GPX4, we overexpressed GPX4 to detect the gene expression changes of YAP and catenin. Overexpression of GPX4 increased the resistance of osteoblasts to compression‐side ferroptosis. When GPX4 is overexpressed, YAP and catenin increased under compressive force (Figure 5F–I) (in Supplementary Material).

#### 3.1.6 GPX4 promoted ferroptosis via the YAP‐TEAD signalling pathway under mechanical compression force

**FIGURE 6:**
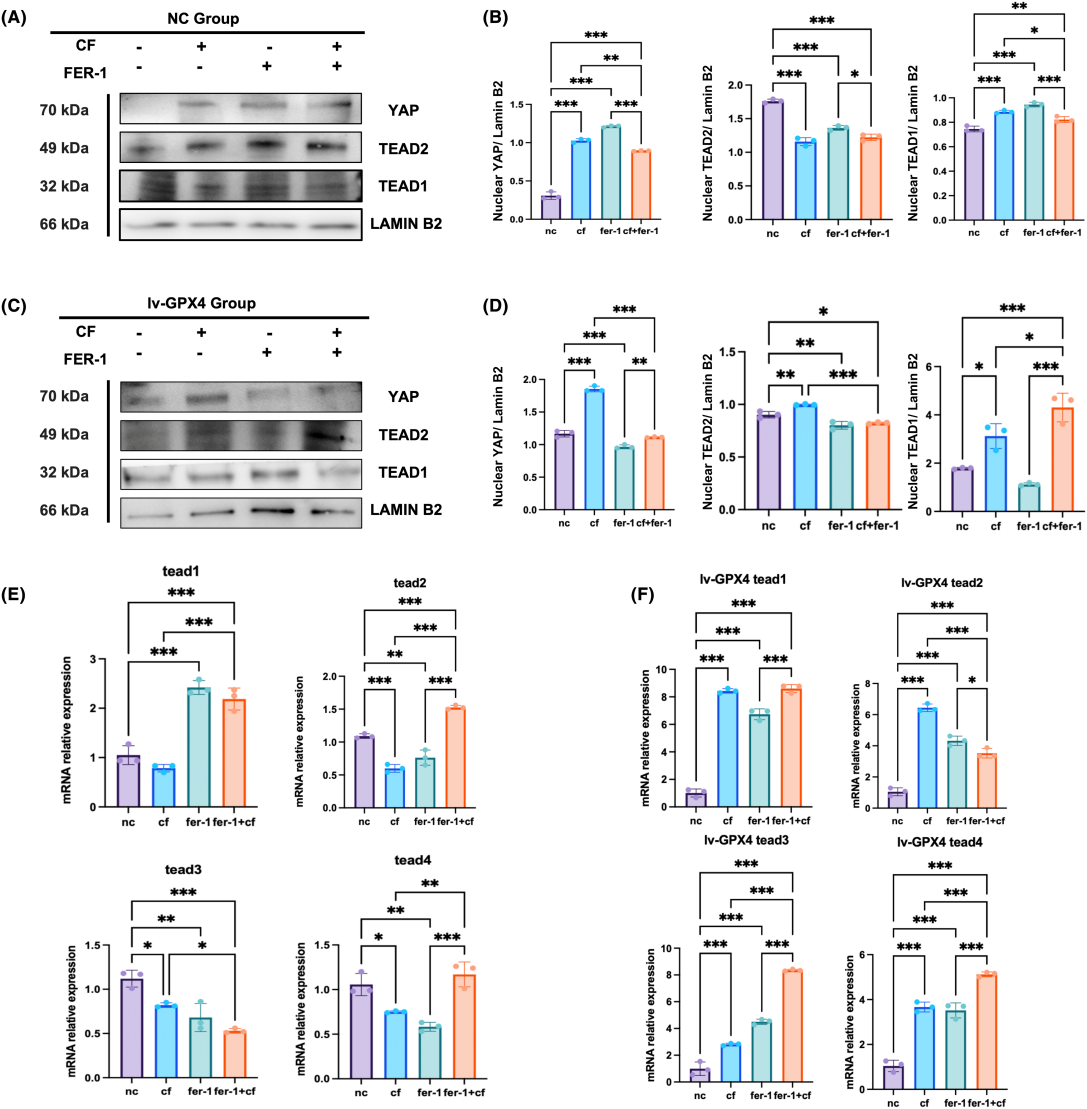
GPX4 played an important role in YAP‐TEAD nuclear transcription. (A) tead1 mRNA level was increased by compression force MC‐3T3 cells, compared with other TEAD family members showing no difference. (B) In lv‐GPX4 cells, pressure promoted TEAD family members' mRNA levels. (C) YAP, TEAD1, and TEAD2 protein expression in MC‐3T3 cells. Fer‐1 promoted YAP transduction from cytoplasm to nuclear. (D) YAP, TEAD1, and TEAD2 protein expression in lv‐GPX4 MC‐3T3 cells. Pressure‐induced YAP transduction. (E) the protein ration of YAP, TEAD1 and TEAD2 in MC‐3T3 cells. (F) The protein ratio of YAP, TEAD1, and TEAD2 in lv‐GPX4 MC‐3T3 cells. A Student's *t*‐test or one‐way ANOVA was conducted to ascertain statistical significance, with all error bars indicating the SD (*n* = 3). *P<0.05, **p ≤ 0.01, ***P<0.001.

**FIGURE 7:**
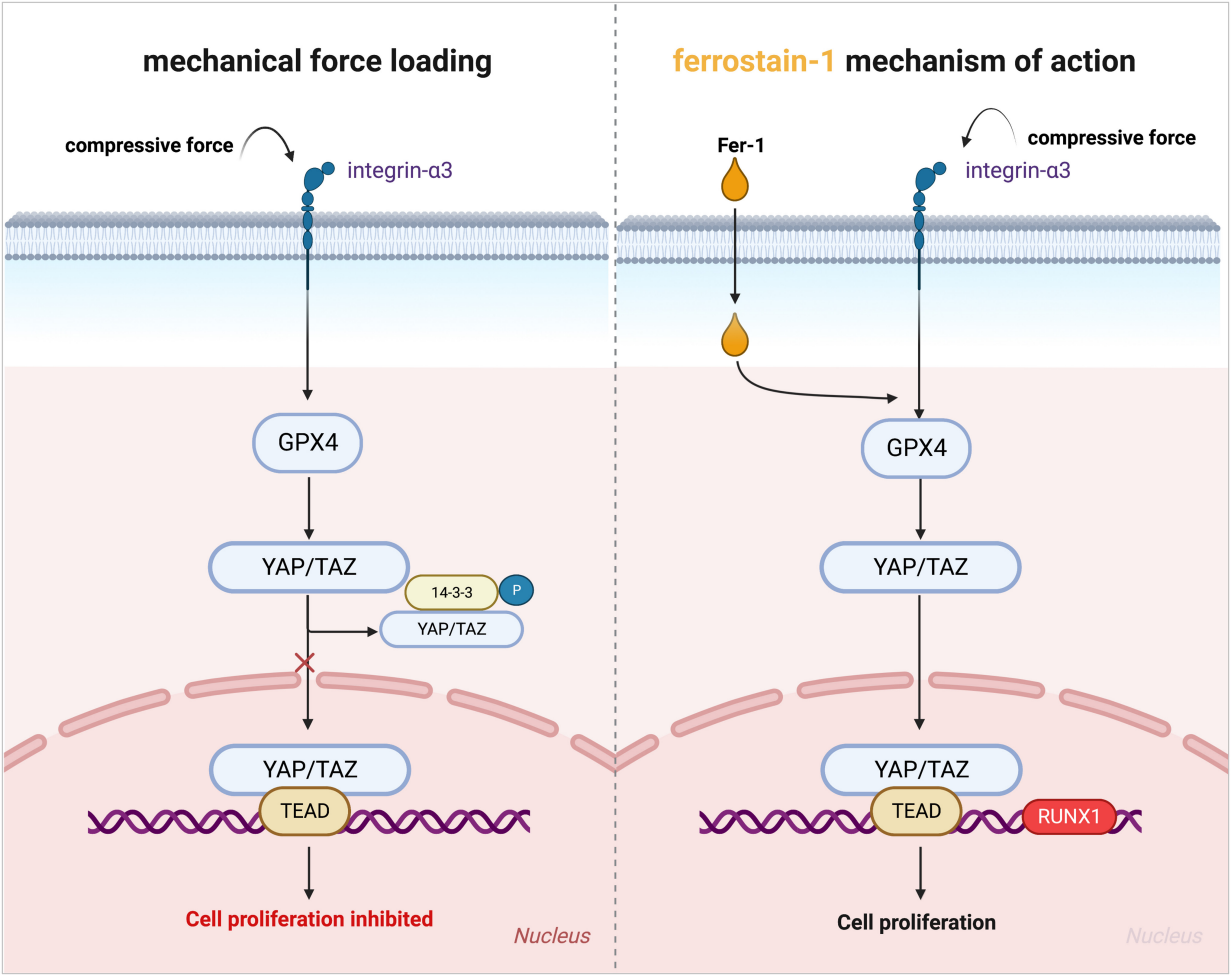
Schematic for the role of compressive force in ferroptosis of MC‐3T3. (1) when compressive force loading on osteoblasts, integrin accepted mechanical signal to conduct to GPX4, following with YAP cell signal activation, as a result, cell proliferation inhibited. (2) fer‐1 treated with compressive force, GPX4 was rescued and induced to inhibit the YAP pathway, followed by cell proliferation.

In the intranuclear targets, TEAD1 and TEAD2 levels increased with compression force stimulation. Also, TEAD1 and TEAD2 levels increased significantly under both GPX4 treatment and compression force loading. The YAP nuclear protein level exhibited a similar increasing trend (Figure 6A,B,E). The compression force inhibited TEAD1 and TEAD2 levels in knockdown GPX4 MC‐3T3 compared with MC‐3T3. Meanwhile, Fer‐1 promoted TEAD1 and TEAD2 levels in MC‐3T3, whereas lv‐GPX4 cells exhibited the opposite trend (Figure 6C,D,F). Fer‐1's stimulation of the YAP‐TEAD pathway, which transmitted the signal from the cytoplasm to the nucleus, was revealed by the results, and it was found to have a potential effect on ferroptosis in MC‐3T3 (Figure 7A,B).

## 4 DISCUSSION

Osteoblast regeneration failure is the hallmark of alveolar bone resorption due to orthodontic tooth movement in a clinic setting. Tooth movement is the balance between osteoblasts and osteoclasts, which is associated with optimal orthodontic force. Imbalanced mechanical stress, including stretch and tensile, leads to bone destruction. Researchers are focusing more on transforming mechanical signals into biological signals and controlling the signalling pathway. To date, numerous investigations have concentrated on the YAP‐TEAD signalling pathway, a widely‐used transduction route for mechanical strain. Therefore, we hypothesized that osteoblasts decreased tooth movement via the YAP‐TEAD pathway.

A recently established process of cell death, ferroptosis, is distinguished by a diminished GPX4 concentration and oxidative harm. In this study, we performed compression force loading and stretch to mimic tooth movement on both sides and observed differences between the two areas. Ferroptosis occurred earlier on the compression force side than on the stretch side for about more than 10 hours. After MC‐3T3 treatment with a ferroptosis agonist (Fer‐1) and an inhibitor (erastin), GPX4 and ACSL4 levels exhibited reversed trends, which proved that ferroptosis played a significant role in tooth movement on the compression force side. This result suggested that mechanical compression force inhibited erastin‐induced ferroptosis. Fer‐1 was found to have a remarkable effect on the expression of RUNX2 and OPN. Additionally, we discovered that Fer‐1 promoted bone regeneration and prevented osteoblasts from ferroptosis, especially on GPX4 knockdown cells that induce ferroptosis. It is claimed that GPX4 plays a critical part in ferroptosis, and has a contrary function in the same process.^28^

In this study, GPX4 levels deceased with compression force loading, implying a potential association between GPX4 and mechanical compression force. Hence, GPX4 knockdown with a virus was performed to confirm the significant role of GPX4 in compression force‐induced ferroptosis. The findings indicated that the downregulated GPX4 affected the osteoblast response to mechanical compression force.

Mechanically mediated cells are the site of YAP‐TEAD channels, and the Hippo pathway integrates a variety of signals via the YAP and TAZ activities to manage essential cellular operations. YAP and TAZ are essential regulators of transcription without DNA‐binding domains, often engaging with DNA‐binding proteins to control transcriptional activity. Upon activation, the Hippo pathway restricts tissue growth and cell proliferation by phosphorylating and inhibiting YAP/TAZ. Isolating phosphorylated YAP/TAZ in the cytoplasm through protein interaction, it is then degraded by the ubiquitin‐proteasome system. Conversely, when the Hippo pathway is deactivated, YAP/TAZ is dephosphorylated and sent to the nucleus, where it binds to pigmentation factors, thus inducing transcriptional programs essential for cell proliferation, survival, and migration. YAP/TAZ is also functionally necessary to differentiate MSC with ECM stiffness.^22^, ^26^, ^27^

Instead, activating YAP expression overrides the physical constraints on the behavior of dictating cells. The loss of YAP and TAZ in osteoblasts disrupts the integrity of the skeletal system, reduces bone formation, and improves bone resorption. The expression of osteogenesis and collagen‐related genes is diminished when YAP/TAZ is depleted or YAP/TAZ‐TEAD is inhibited acutely. The composition of the ECM determines its rigidity, which in turn impacts the nuclear localization of YAP/TAZ. This means that YAP/TAZ can regulate the ECM in response to mechanical forces, facilitating a delicate adaptation process between the ECM and the cells.^21^ Our research demonstrated a correlation between YAP‐TEAD activation and ferroptosis, thus demonstrating YAP/TAZ's essential part in the suppression of ferroptosis.^29^

A recent study revealed that mechanical load is a critical factor in sustaining bone equilibrium by controlling bone remodeling. Bone cells are the most plentiful cell type embedded in the mineralized bone matrix and are the primary mechanical sensors that regulate bone remodeling in reaction to mechanical forces. The mechanical sensing capabilities of bone cells activate various receptors and distinct intracellular signaling pathways. YAP/TAZ is regulated by mechanical and cytoskeletal signals and acts as a mechanical converter to control cell fate in response to the characteristics of the cellular microenvironment.^30^ Interaction between YAP/TAZ and osteoblast signaling pathways, such as the β/bone morphogenetic protein transformation and Wnt/β‐catenin signaling, is observed.^31^

Our research revealed that, following GPX4 knockdown, compression force stimulated the Hippo‐YAP signalling pathway, thus hindering GPX4 proliferation; however, Fer‐1 treatment prevented cell death and inhibited the Hippo‐YAP pathway. Thus, GPX4 might be a key downstream target of YAP in mechanical compression force‐induced ferroptosis.

We also examined the nuclear targets of the YAP pathway and TEAD1‐4. On mechanical loading, TEAD1 and TEAD2 were both activated and promoted transcription in MC‐3T3. The Fer‐1 treatment‐induced TEAD1 and TEAD2 transcription remarkably. Thus, we indicated that GPX4 promoted the YAP‐TEAD pathway transcription in the nucleus. After reducing the GPX4 level, we found that compression force decreased TEAD1 and TEAD2 levels. Meanwhile, Fer‐1 increased TEAD1 and TEAD2 transduction, indicating that Fer‐1 might promote YAP transcription responding to mechanical compression force in GPX4‐dependent cells.

In summary, this study described the underlying mechanism of mechanical compression force‐induced ferroptosis in osteoblasts, especially in ferroptosis associated with the YAP‐TEAD pathway. Moreover, GPX4 played a vital role in compression force‐induced ferroptosis. This finding might provide insight into potential therapy in orthodontic treatment to prevent bone destruction (Figure 7A,B).

## 5 CONCLUSIONS

Ferroptosis occurs because of the movement of the orthodontic teeth. It first affects the compression force side and then the stretch side within 4 h.GPX4's consequence of alveolar bone loss is inhibition by Fer‐1, whereas compression force‐side loss in lateral alveolar bone activates the Hippo‐YAP pathway, which is based on GPX4.

## AUTHOR CONTRIBUTIONS

**Wang Mengjia:** Conceptualization (equal); investigation (equal); writing – original draft (lead). **Ji Jun:** Formal analysis (equal); project administration (supporting); supervision (equal). **Zhang Xin:** Data curation (equal); resources (equal). **Zhang Jiahao:** Formal analysis (equal); investigation (equal); writing – review and editing (equal). **Guo Jie:** Funding acquisition (supporting); project administration (equal); supervision (equal).

## FUNDING INFORMATION

The National Natural Science Foundation of China (Grant No. 81970964, 82370999) generously funded this study.

## CONFLICT OF INTEREST STATEMENT

The authors proclaim that the research was done without any commercial or financial ties that could be seen as a possible conflict of interest.

## Supporting information


Appendix S1.

Figure Caption.

## Data Availability

All data are contained within the Article, Supplementary Information, or available from the authors upon request.

## References

[1] Almeida M . Aging mechanisms in bone. Bonekey Rep. 2012;1:102. 23705067 10.1038/bonekey.2012.102 PMC3659822

[2] Arai M , Shibata Y , Pugdee K , Abiko Y , Ogata Y . Effects of reactive oxygen species (ROS) on antioxidant system and osteoblastic differentiation in MC3T3‐E1 cells. IUBMB Life. 2007;59(1):27‐33. 17365177 10.1080/15216540601156188

[3] Hendrickx G , Boudin E , Van Hul W . A look behind the scenes: the risk and pathogenesis of primary osteoporosis. Nat Rev Rheumatol. 2015;11(8):462‐474. 25900210 10.1038/nrrheum.2015.48

[4] Kim KH , Lee MS . Autophagy‐a key player in cellular and body metabolism. Nat Rev Endocrinol. 2014;10(6):322‐337. 24663220 10.1038/nrendo.2014.35

[5] Jeney V . Clinical impact and cellular mechanisms of iron overload‐associated bone loss. Front Pharmacol. 2017;8:77. 28270766 10.3389/fphar.2017.00077 PMC5318432

[6] Medeiros DM . Copper, iron, and selenium dietary deficiencies negatively impact skeletal integrity: a review. Exp Biol Med (Maywood). 2016;241(12):1316‐1322. 27190269 10.1177/1535370216648805 PMC4950270

[7] Weaver CM , Gordon CM , Janz KF , et al. The National Osteoporosis Foundation's position statement on peak bone mass development and lifestyle factors: a systematic review and implementation recommendations. Osteoporos Int. 2016;27(4):1281‐1386. 26856587 10.1007/s00198-015-3440-3 PMC4791473

[8] Gittoes N . Progress and problems in bone and mineral disorders. Eur Endocrinol. 2017;13(1):19‐20. 29632601 10.17925/EE.2017.13.01.19 PMC5813440

[9] Cheng Q , Zhang X , Jiang J , et al. Postmenopausal iron overload exacerbated bone loss by promoting the degradation of type I collagen. Biomed Res Int. 2017;2017:1345193. 28620614 10.1155/2017/1345193 PMC5460413

[10] Guggenbuhl P , Fergelot P , Doyard M , et al. Bone status in a mouse model of genetic hemochromatosis. Osteoporos Int. 2011;22(8):2313‐2319. 20976594 10.1007/s00198-010-1456-2

[11] Simao M , Camacho A , Ostertag A , et al. Correction: iron‐enriched diet contributes to early onset of osteoporotic phenotype in a mouse model of hereditary hemochromatosis. PLoS One. 2019;14(4):e0216377. 31034507 10.1371/journal.pone.0216377 PMC6488066

[12] Doyard M , Chappard D , Leroyer P , Roth MP , Loréal O , Guggenbuhl P . Decreased bone formation explains osteoporosis in a genetic mouse model of hemochromatosiss. PLoS One. 2016;11(2):e0148292. 26829642 10.1371/journal.pone.0148292 PMC4734777

[13] Yang WS , SriRamaratnam R , Welsch ME , et al. Regulation of ferroptotic cancer cell death by GPX4. Cell. 2014;156(1–2):317‐331. 24439385 10.1016/j.cell.2013.12.010 PMC4076414

[14] Mao C , Liu X , Zhang Y , et al. DHODH‐mediated ferroptosis defence is a targetable vulnerability in cancer. Nature. 2021;593(7860):586‐590. 33981038 10.1038/s41586-021-03539-7 PMC8895686

[15] Simao M , Camacho A , Ostertag A , et al. Iron‐enriched diet contributes to early onset of osteoporotic phenotype in a mouse model of hereditary hemochromatosis. PLoS One. 2018;13(11):e0207441. 30427936 10.1371/journal.pone.0207441 PMC6241130

[16] Callaway DA , Jiang JX . Reactive oxygen species and oxidative stress in osteoclastogenesis, skeletal aging and bone diseases. J Bone Miner Metab. 2015;33(4):359‐370. 25804315 10.1007/s00774-015-0656-4

[17] Ikebuchi Y , Aoki S , Honma M , et al. Coupling of bone resorption and formation by RANKL reverse signalling. Nature. 2018;561(7722):195‐200. 30185903 10.1038/s41586-018-0482-7

[18] Lu M , Liu Y , Shao M , Tesfaye GC , Yang S . Associations of iron intake, serum iron and serum ferritin with bone mineral density in women: the National Health and nutrition examination survey, 2005‐2010. Calcif Tissue Int. 2020;106(3):232‐238. 31754762 10.1007/s00223-019-00627-9

[19] Yang WP , Chang HH , Li HY , et al. Iron overload associated endocrine dysfunction leading to lower bone mineral density in thalassemia major. J Clin Endocrinol Metab. 2020;105(4):e1015‐e1024. 10.1210/clinem/dgz309 31907538

[20] Angelopoulos NG , Goula AK , Papanikolaou G , Tolis G . Osteoporosis in HFE2 juvenile hemochromatosis. A case report and review of the literature. Osteoporos Int. 2006;17(1):150‐155. 15997423 10.1007/s00198-005-1920-6

[21] Yang WH , Chi JT . Hippo pathway effectors YAP/TAZ as novel determinants of ferroptosis. Mol Cell Oncol. 2020;7(1):1699375. 31993503 10.1080/23723556.2019.1699375 PMC6961671

[22] Wu J , Minikes AM , Gao M , et al. Intercellular interaction dictates cancer cell ferroptosis via NF2‐YAP signalling. Nature. 2019;572(7769):402‐406. 31341276 10.1038/s41586-019-1426-6 PMC6697195

[23] Xiao W , Beibei F , Guangsi S , et al. Iron overload increases osteoclastogenesis and aggravates the effects of ovariectomy on bone mass. J Endocrinol. 2015;226(3):121‐134. 26116610 10.1530/JOE-14-0657

[24] Yamasaki K , Hagiwara H . Excess iron inhibits osteoblast metabolism. Toxicol Lett. 2009;191(2–3):211‐215. 19735707 10.1016/j.toxlet.2009.08.023

[25] Yang J , Zhang J , Ding C , Dong D , Shang P . Regulation of osteoblast differentiation and iron content in MC3T3‐E1 cells by static magnetic field with different intensities. Biol Trace Elem Res. 2018;184(1):214‐225. 29052173 10.1007/s12011-017-1161-5 PMC5992240

[26] Yang WH , Ding CKC , Sun T , et al. The hippo pathway effector TAZ regulates Ferroptosis in renal cell carcinoma. Cell Rep. 2019;28(10):2501‐2508.e4. 31484063 10.1016/j.celrep.2019.07.107 PMC10440760

[27] Yang WH , Huang Z , Wu J , Ding CKC , Murphy SK , Chi JT . A TAZ‐ANGPTL4‐NOX2 axis regulates Ferroptotic cell death and Chemoresistance in epithelial ovarian cancer. Mol Cancer Res. 2020;18(1):79‐90. 31641008 10.1158/1541-7786.MCR-19-0691 PMC6942206

[28] Lei G , Mao C , Yan Y , Zhuang L , Gan B . Ferroptosis, radiotherapy, and combination therapeutic strategies. Protein Cell. 2021;12(11):836‐857. 33891303 10.1007/s13238-021-00841-y PMC8563889

[29] Gao R , Kalathur RKR , Coto‐Llerena M , et al. YAP/TAZ and ATF4 drive resistance to Sorafenib in hepatocellular carcinoma by preventing ferroptosis. EMBO Mol Med. 2021;13(12):e14351. 34664408 10.15252/emmm.202114351 PMC8649869

[30] Zarka M , Etienne F , Bourmaud M , et al. Mechanical loading activates the YAP/TAZ pathway and chemokine expression in the MLO‐Y4 osteocyte‐like cell line. Lab Investig. 2021;101(12):1597‐1604. 34521992 10.1038/s41374-021-00668-5

[31] Regard JB , Zhong Z , Williams BO , Yang Y . Wnt signaling in bone development and disease: making stronger bone with Wnts. Cold Spring Harb Perspect Biol. 2012;4(12):a007997. 23209148 10.1101/cshperspect.a007997 PMC3504445

