# Supplementary figures and images for: GPX4‐mediated bone ferroptosis under mechanical stress decreased bone formation via the YAP‐TEAD signalling pathway
Source: J Cell Mol Med. 2024 Mar 17;28(7):e18231. doi: 10.1111/jcmm.18231 (PMC10945084; doi:10.1111/jcmm.18231)

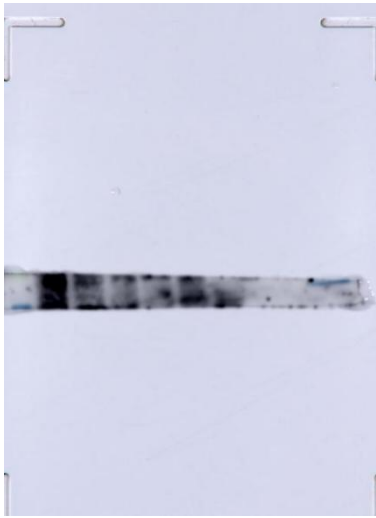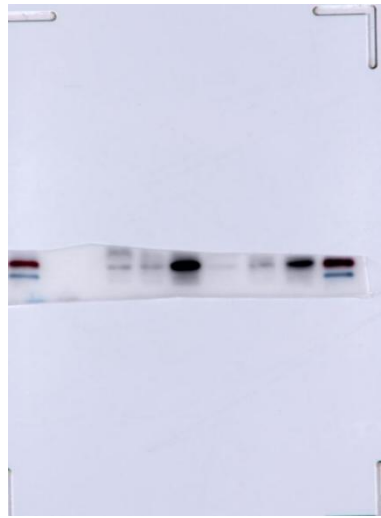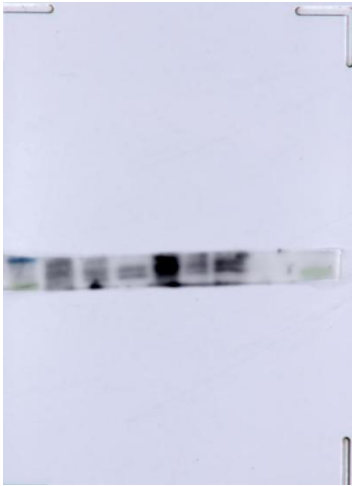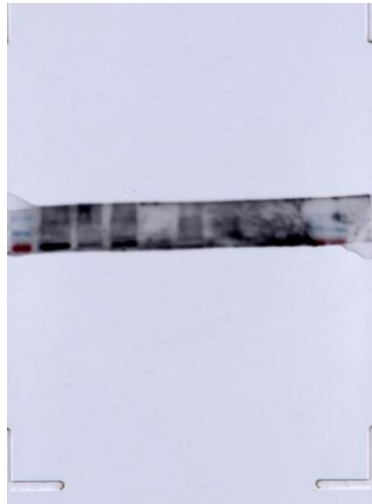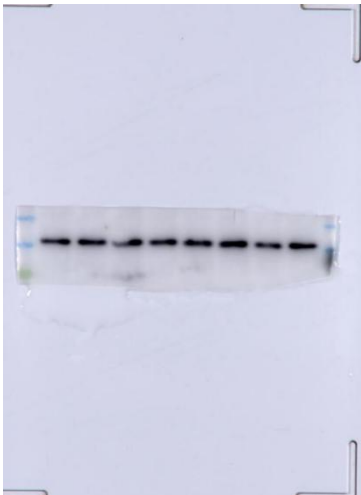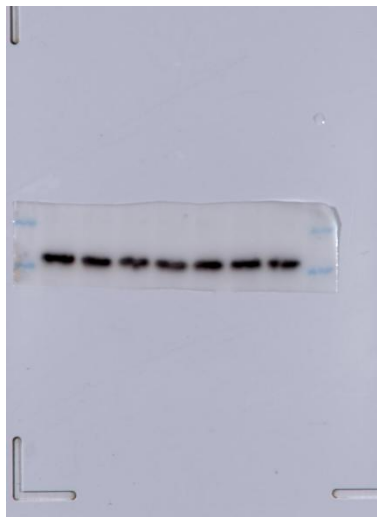

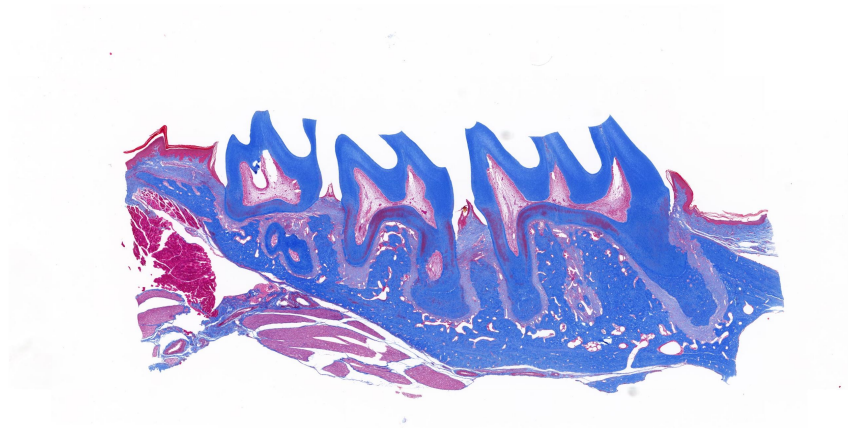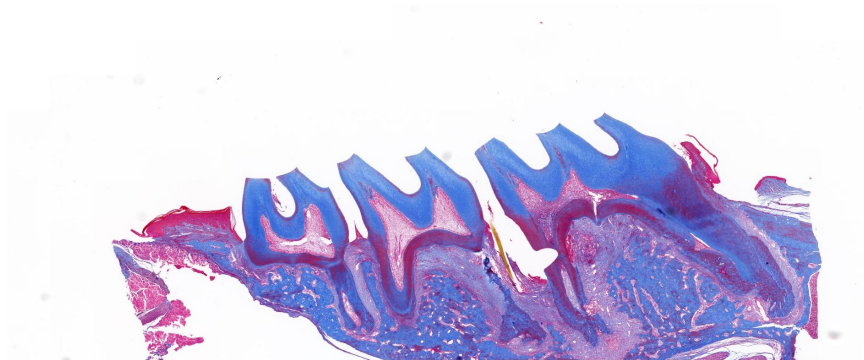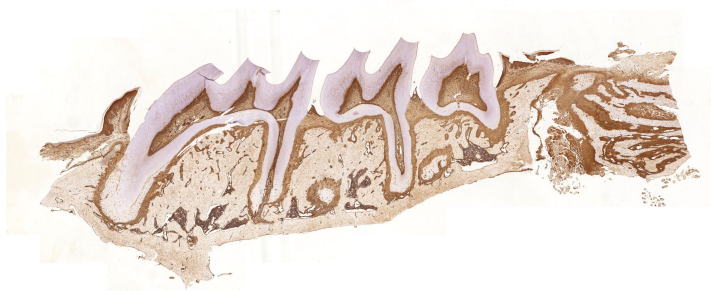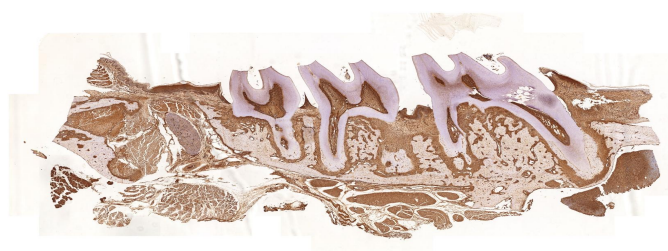

Supplement: Supplementary file 1 — Appendix S1. [file JCMM-28-e18231-s001.pdf]
