# Supplementary material for: GPX4‐mediated bone ferroptosis under mechanical stress decreased bone formation via the YAP‐TEAD signalling pathway
Source: J Cell Mol Med. 2024 Mar 17;28(7):e18231. doi: 10.1111/jcmm.18231 (PMC10945084; doi:10.1111/jcmm.18231)
Supplement: Supplementary file 2 — Figure Caption. [file JCMM-28-e18231-s002.docx]

Appendix S1:

Duplication of western blot including all the strips.
